# Seasonal intensification and trends of rogue wave events on the US western seaboard

**DOI:** 10.1038/s41598-019-41099-z

**Published:** 2019-03-14

**Authors:** A. D. Cattrell, M. Srokosz, B. I. Moat, R. Marsh

**Affiliations:** 1 Fluid Structure Interactions, Engineering and the Environment, Boldrewood Innovation Campus, University of Southampton, Southampton, SO17 1BJ UK; 2 National Oceanography Centre, University of Southampton Waterfront Campus, European Way, Southampton, SO14 3ZH UK; 3 Ocean and Earth Science, University of Southampton, National Oceanography Centre Southampton, Southampton, SO14 3ZH UK

## Abstract

Studies of changes in wave climate typically consider trends in sea state statistics, such as the significant wave height. However, the temporal variability of individual rogue waves, which pose a hazard to users of the sea and coastal environment has not been investigated. We use time series of continuous surface elevation over 124–270 months (spanning 1994–2016), from 15 wave buoys along the US western seaboard, to investigate regional trends in significant wave height and individual rogue waves. We find high spatial variability in trends in significant wave height and rogue waves across the region. Rogue wave occurrence displays a mostly decreasing trend, but the relative height – or severity – of the waves is increasing. We also identify seasonal intensification in rogue waves with increased rogue wave occurrence, of higher severity, in the winter than in the summer. Therefore, the common practice of stating a single occurrence likelihood for an ocean basin is not valid. In addition, the buoy data show that the magnitude and significance of trends in significant wave height increases towards higher percentiles, supporting previous findings.

## Introduction

Global mean significant wave height has increased over the past 30 years^1–3^. Young *et al*.^3^, analysed satellite altimetry from 1985–2008 that indicated weak trends in monthly global mean significant wave height (*H*_*S*_) and indicated that the largest waves (90^th^ and 99^th^ percentile *Hs*) have increased in amplitude over this period. This is also true for waves along the highly populous western seaboard of the United States of America. These were shown to be increasing, with 90^th^ and 99^th^ percentile of mean monthly *Hs* increasing by over 1% per year, over a 23 year period^3^. *In-situ* measurements yielded negative trends offshore of Washington and Oregon, and positive offshore of California, all of which were of small magnitude^4^. Similarly, neutral or weakly decreasing trends in *Hs* have also been seen in other observation-based studies^5,6^. In addition, decreasing trends in mean peak wave period (*Tp*) were seen from 1992 to 2012 for most of the mid-latitude North Pacific^6^.

**Figure 1:**
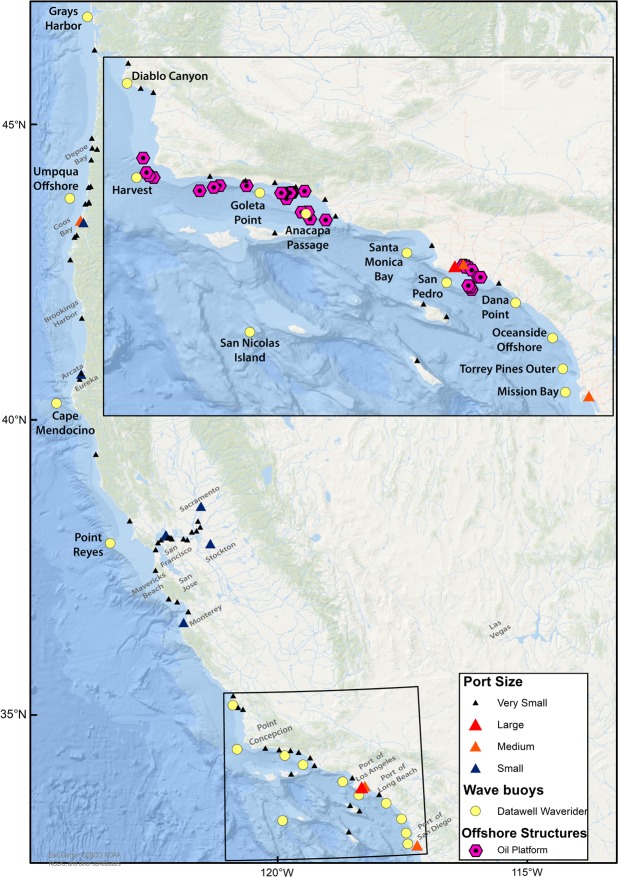
Overview map of the United States western seaboard. The locations of the 15 Datawell Waverider buoys are shown (yellow points), along with ports (triangle markers) and offshore structures (pink hexagons). ESRI Basemap Sources: Esri, GEBCO, NOAA, National Geographic, DeLorme, HERE, Geonames.org, and other contributors.

Rogue waves are transient individual waves larger than expected for the surrounding sea state, and are known to occur on the US western seaboard^7,8^. The region has a population of over 50 million people with significant coastal infrastructure. Ports in the region handle 49% of the total U.S. containerised trade and Port of Los Angeles and Port of Long Beach together make Southern California the largest gateway for U.S. containerised imports^9^. In addition, there is a high volume of tanker, bulk carrier, Roll On–Roll Off, passenger, and fishing activity focused around the ports in the region (Fig. 1). Vessels also service the offshore structures deployed to support the Californian oil and gas industry^10^, as well as test beds for renewable energy devices (Fig. 1). Rogue waves at the shoreline have fatally swept people out to sea at Point Reyes, Maverick’s beach, and Arcata, in California and Depoe Bay, Oregon^11^. Rogue wave-ship collisions have resulted in fatalities, cargo loss, and ships foundering^12^. In the region, the NOAA research vessel R/V *Ballena* capsised off Point Conception, California^13^. In 2006, M/V *Westwood Pomona* encountered a rogue wave off Port of Coos Bay, injuring one and damaging the vessel. In Brookings Harbor, Oregon, four waves caused ship damage^11^.

Rogue waves are differentiated from normal waves as waves of height (*h*) greater than 2*Hs* $(\frac{h}{Hs} > 2)$, or with a crest height (*Cr*) greater than 1.25*Hs* $(\frac{Cr}{Hs} > 1.25)$^14^. Previous research on rogue waves evaluates their occurrence likelihood from compiled datasets across multiple locations^7^, and typically give one statistic for their occurrence^15^. Recent work has expanded this to consider their spatial variability along the US western seaboard^8^, and seasonality^16^, but the interannual temporal variability of the phenomenon is unknown. Here we provide a detailed temporal analysis of the wave record from 15 Datawell Waverider buoys along the US western seaboard (Fig. 1), managed by the Coastal Data Information Program (CDIP). They were selected as they store the raw surface displacement data and provide a uniform sampling platform with a duration of over 10 years. First, we analyse whether there is a statistically significant monotonic trend in the monthly binned sea state characteristics over sampling periods of 124–270 months and compare to the findings of previous studies. Temporal variability of individual rogue wave events, and their height and relative abnormality are assessed on both a decadal and seasonal scale for the first time.

## Results

### Are Wave Heights Increasing?

**Figure 2:**
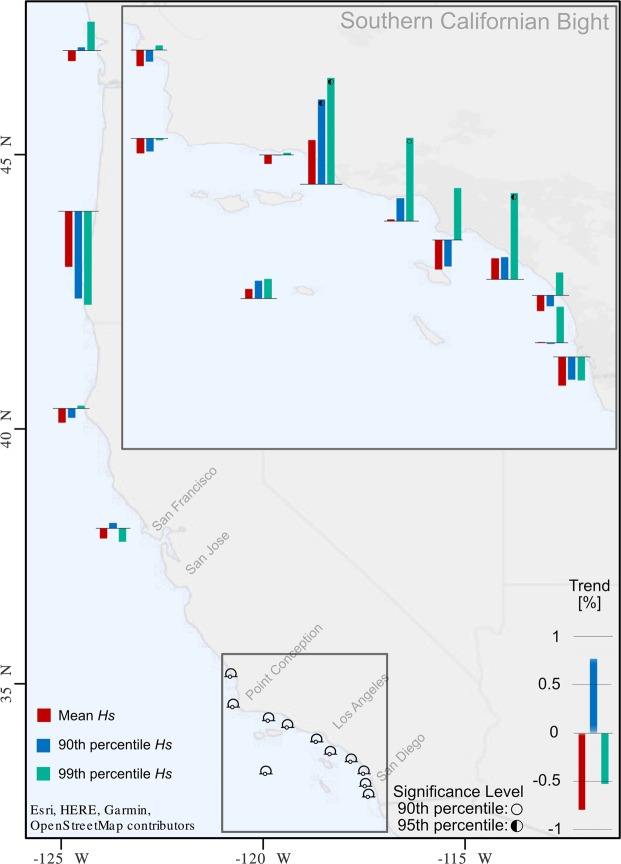
Trend in the mean (red), 90^th^ percentile (blue), and 99^th^ percentile (green) of significant wave height as a percentage of the location’s time series mean. Empty and half-filled circles indicate the 90^th^ and 95^th^ levels of statistical significance, respectively, deemed by the seasonal Mann-Kendall test.

The mean *Hs* over the sampling period of the buoys show small decreasing trends of less than 0.5% yr^−1^ for most buoys; although these are not statistically significant (Red bars – Fig. 2) they are similar to what is seen in satellite data^3^. The trends from buoys in the Southern Californian Bight are slightly more complex, with four buoys displaying an increasing trend. These four buoys include the only statistically significant buoy and displays an increase in the 90^th^ percentile of *Hs* (Blue bars – Fig. 2). Elsewhere buoys show a reduction in the 90^th^ percentile of *Hs*. Trends in 99^th^ percentile of *Hs* (Green bars - Fig. 2) have the largest magnitudes. Trends are mostly positive, with magnitudes around 0.02 m yr^−1^, and three buoys have statistically significant trends. In Oregon, Umpqua Offshore shows a reduction of 0.004 m yr^−1^ (0.96% y^−1^). In California, Anacapa Passage, Santa Monica Bay, San Pedro, and Dana Point show increases of 0.009 to 0.021 m yr^−1^ (0.5–1.1%y^−1^). The magnitude of significant wave height trend increases at extreme values (90^th^ and 99^th^ percentiles) supporting previous findings^3^; however, the sign of the trends are more spatially variable than seen in 2° × 2° satellite data^3^.

Trends in the mean wave crest heights are weak and none are statistically significant. Trends in the 99^th^ percentile of crest height are stronger, with the three buoys that showed statistically significant results for the 99^th^ percentile of *Hs* also having significantly increasing mean crest heights (TABLE S1). The trend in mean peak wave period (*Tp)* and mean zero-crossing wave period (*Tz*) are reducing as projected by Hemer *et al*.^17^; however, in locations sheltered from the dominant wave direction – inshore buoys between point conception and San Diego - the mean *Tz* is increasing. Here the buoys are more wind-sea dominated and respond to changing wind forcing^3^, whereas more exposed buoys are swell dominated, and changes in wind-sea are less discernible. The magnitudes of the trends are less than 0.3% y^−1^. Furthermore, the distribution of energy over frequency components, as described by the spectral bandwidth parameter, *ν* (*see Methods*), is also reducing at most locations (TABLE S1).

### Are Rogue Wave occurrences increasing?

**Figure 3:**
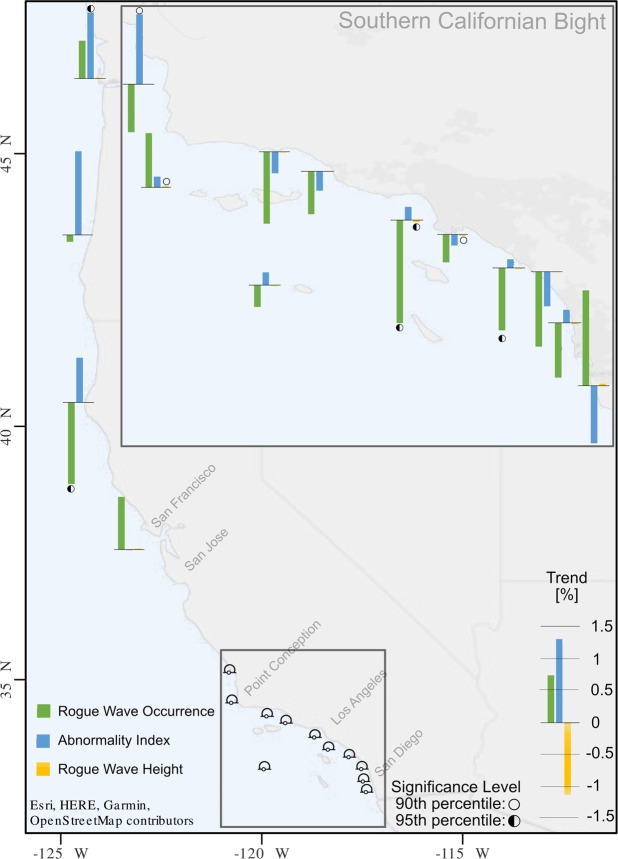
Trends in rogue wave occurrence (green), Excess Abnormality Index (*Excess AI;* blue), and rogue wave height (yellow), as a percentage of the mean value of the time series. Empty and half-filled circles indicate the 90^th^ and 95^th^ significance levels, respectively, as determined by a seasonal Mann-Kendall test.

Rogue wave occurrence is variable on an interannual timescale, and there is considerable spatial variability within the region (Green bars – Fig. 3). Rogue wave occurrence is reducing within the Southern Californian Bight, but elsewhere trend and magnitude vary. As a percentage of the average occurrence at each location, rogue wave occurrences are increasing at 1.5% y^−1^ at the most southerly buoy, Mission Bay, CA. Although larger increasing trends of 0.6, 0.81, and 0.84 % y^−1^ are seen in the north at Grays Harbor, Point Reynes, and Harvest, respectively, these are not statistically significant. Buoys that do have significant trends are ones with a reduction in rogue wave occurrence: −1.3% y^−1^ at Cape Mendocino, CA.; −1.6% y^−1^ at Santa Monica Bay, CA.; and −1% y^−1^ at Dana Point CA. (Fig. 3).

We have discussed the trends in occurrence of rogue waves, but the temporal variability of the size of the rogue waves is unknown and is important in determining their impact on the coastal marine industry. The Abnormality Index (*AI*) gives a useful metric for how large a rogue is with respect to the background sea (*see Methods*). Here we investigate the *excess AI*, defined as $\{(\frac{{H}_{rogue}}{{H}_{s}})-2\}$ for a wave height rogue, or $\{(\frac{C{r}_{rogue}}{{H}_{s}})-1.25\}$ for a crest height rogue. Most buoys show minimal change in *excess AI*, but Grays Harbor, WA. and Diablo Canyon, CA. show statistically significant increases over time of greater than 1% y^−1^ (Blue bars – Fig. 3). At these locations rogue waves are getting monotonically larger with time, relative to the background sea (*Hs*), and therefore more hazardous to maritime industry at Grays harbor, and coastal infrastructure such as the nuclear power plant at Diablo Canyon. There is however, minimal temporal variability in the absolute height of rogue waves (Yellow bars – Fig. 3). As a percentage of the mean rogue wave height, trends are all less than 0.02% y^−1^.

The frequency and intensity of the weather systems in the region are connected to the Aleutian Low^5,18^, and indirectly to the El Niño Southern Oscillation (ENSO)^19^ and the Arctic Oscillation^20^. Signals of increased *Hs* and *Tp* during the 1997–1998 and 2015–2016 El Niño years are seen in the dataset, and previous analyses^21^. However, we find no discernible link between the interannual variations in rogue wave occurrence and climate variability indices.

The analysis is aimed at assessing the interannual variability of rogue waves and determining monotonic linear trends over the wave buoy observational period. It does not necessarily indicate that the trends are a result of global climate change as interannual climate variations have long-term oscillations that will influence the wave climate on a longer time scale than the data record presented here. Furthermore, the length of this dataset is too short to differentiate a steady trend from an accelerating trend.

### Seasonal Intensification of Rogue Waves

**Figure 4:**
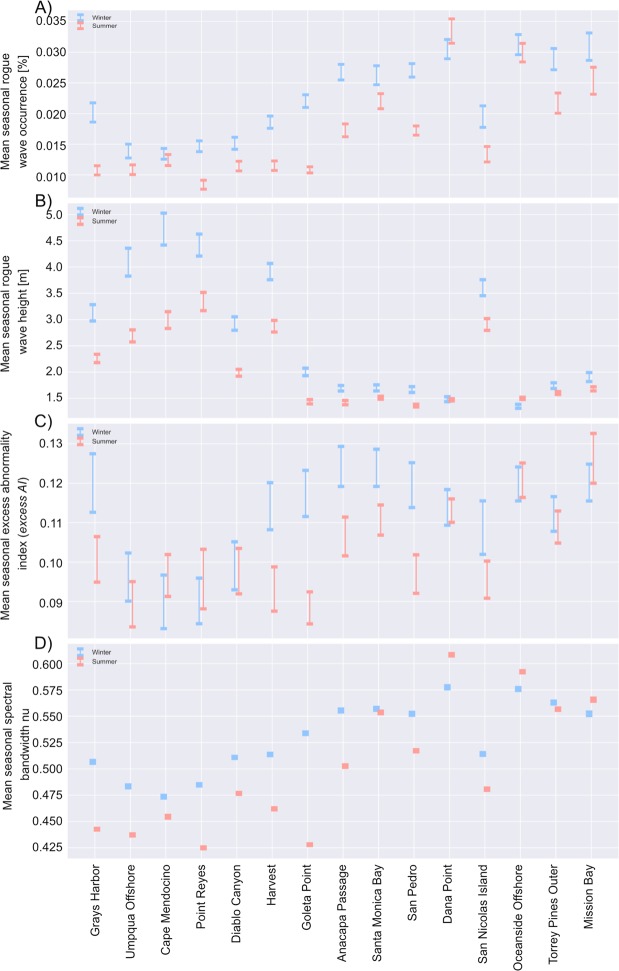
Mean Winter (blue) and Summer (red) values for each buoy, from north to south, for (**A**) the rogue wave occurrence, (**B**) rogue wave height, (**C**) Abnormality Index (*AI*), and (**D**) spectral bandwidth parameter Nu. Error bars denote two standard error about the mean.

The only previous research on seasonality showed an increase in rogue wave occurrence in the both summer and winter^16^. In contrast., we find that rogue waves are more prevalent in the winter months (December-January-February, blue - Fig. 4A) than summer (June-July-August, red). Grays Harbor, WA. and Goleta Point, CA. have almost double the probability of rogue wave occurrence in winter compared to summer. The exceptions are the buoys with the lowest wave energy, Dana Point, CA., and Oceanside CA. The contrast to previous findings is possibly due to a different choice of rogue wave definition and analysis approach, differing record lengths and the inclusion of buoys from different regions, as compared to Imai (2010)^16^. Our analysis shows the need to determine seasonal occurrence statistics for individual rogue waves rather than just annual averages.

Winter also yields larger rogue waves than summer (Fig. 4B), with exposed northern locations displaying the largest seasonal variation, with almost a 2 m variation in average seasonal height at Cape Mendocino. In contrast, rogue waves in the sheltered locations of the Southern Californian Bight have a seasonal variation in height of less than 0.5 m. Seasonality in the absolute height of rogue waves is expected because rogue wave height is a function of *Hs*, which is well known to vary seasonally. During boreal spring and summer, high atmospheric pressure dominates and the waves are forced by north-westerly winds and storms in the southern hemisphere, resulting in lower wave energy conditions^17,18,22^. Outside of this, the wave climatology is dominated by mid-latitude North Pacific extra-tropical cyclones, Eastern Pacific tropical storms, and local winds^22–26^, resulting in large seas, dominated by swell^22,27^. Thus, greater seasonality can be seen at exposed and swell dominated locations, and the distribution of rogue wave height across the region mirrors that of *Hs* (Supplementary Table S1).

*Excess AI* gives an indicator of the size of the rogues compared to *Hs* and hence unaffected by seasonality in *Hs*. Half the buoys locations show statistically significant seasonality in *Excess AI* (Fig. 4C), revealing rogue waves to be ‘more rogue’ during the winter months than during the summer. There are therefore a greater number of rogue waves in winter, and those waves are more severe. The cause of this seasonality may be related to the variation of spectral bandwidth (Fig. 4D), which displays clear seasonality across the region, with bandwidth increasing in the winter months. This mirrors the seasonal distribution in seasonal rogue wave occurrence (Fig. 4A), and seasonal *excess AI* (Fig. 4C). Previous work has shown a rogue waves occur in seas with a large spectral bandwidth^8,28^. A regression of spectral bandwidth trend and rogue wave occurrence trend for each of the buoy locations displays a linear relationship between decreasing rogue wave occurrence and decreasing bandwidth.

## Conclusions

The interannual variability of monthly binned significant wave heights in the US western seaboard shows weak decreasing trends in the mean. In line with previous studies, the magnitude and significance of the trend increased at higher percentiles of *Hs*, but the present analysis indicates high spatial variability in the trend’s magnitude and sign throughout the region. For the first time, we show that rogue waves have interannual temporal variability, and the magnitude and sign of the trend varies over the region, with only the Southern Californian Bight showing a consistent negative trend, and is likely a reflection of the physical complexity of the region. Some locations show that the rogues are getting larger relative to the background sea; however, there is no trend in absolute rogue wave height. However, on a seasonal scale, rogue waves show intensification, with rogues being more prevalent and more severe in the winter than the summer. This is likely due to the increase in spectral bandwidth in the winter. The analysis demonstrates that if statistics of rogue waves are to prove useful for shipping and offshore operations, they must account for spatial and temporal variability on seasonal and interannual timescales, rather than simply relying on a single value for an entire region for all times.

## Material and Methods

### Observational data

To perform rogue wave analysis, the individual wave height (*h*) of each wave needs to be known, as well as the significant wave height (*Hs*) of the sea over the standard analysis period of 20-minutes. Individual wave height can be calculated both from fixed platforms and surface following wave buoys. Fixed platforms use visual or acoustic methods of estimating the distance from a fixed point to the free surface, and are subject to inaccuracies due to spray and wave interactions with the structure. Wave buoys are neutrally buoyant and track the free surface and measure wave height using accelerometers. They are subject to inaccuracies due to the restoring force of the mooring so potentially avoiding the true peak of the wave. Here we chose to use wave buoys due to their broad distribution across the region, public data accessibility, and unparalleled continuity of measurement. The majority of wave buoys deployed globally calculate sea state statistics onboard, and do not store the raw surface elevation. As a result, time-series of rogue waves are limited in duration and count to those buoys that do store the raw surface elevation (*η*). The Coastal Data Information Program (CDIP), operated by Scripps Institution of Oceanography, manages a portfolio of wave buoys around the coastlines of the United States and its overseas dependencies. Of these, 15 have a raw surface elevation data duration of at least 10 years and are located in the US western seaboard (Supplementary Table S1). The buoys use accelerometers to measure waves with periods of 1.6–30 s and wave heights up to 40 m with a vertical resolution of 0.01 m.

### Calculating sea state parameters

The data obtained from the buoys is a time series of vertical displacement sampled at a rate of 3.84 Hz but logged at a sampling frequency of 1.28 Hz.

This is split into non-overlapping 20-minute seas, and the sea state parameters are calculated, as outlined in Cattrell *et al*.^8^. *In situ* measurements require substantial quality control (QC), we perform a strict QC procedure, as outlined in Cattrell *et al*.^8^, to obtain a reliable dataset.

Seas are flagged as rogue if they contain a wave or crest height that exceeds a threshold in relation to the significant wave height (*Hs*)^14^:

1 $$\frac{{H}_{\max }}{Hs} > 2\,$$

and/or

2 $$\,\frac{C{r}_{\max }}{Hs} > 1.25$$

where *H*_*max*_ is the maximum zero-crossing wave height, *Cr*_*max*_ is the maximum crest height, and *Hs* is the significant wave height, estimated as four times the standard deviation of the sea surface elevation from a 20-minute observation period.

The Abnormality Index (*AI*) gives a metric to the severity of the rogue waves:

3 $$(\frac{{h}_{rogue}}{Hs})\,{\rm{or}}\,(\frac{C{r}_{rogue}}{Hs})\,$$

For ease of interpretation we normalise the AI and examine the *excess AI*:

4 $$(\frac{{h}_{rogue}}{Hs})-2\,{\rm{or}}\,\,(\frac{C{r}_{rogue}}{Hs})-1.25$$

Spectral bandwidth can be an indicator of the strength of nonlinear focusing^29^ and the spectral bandwidth parameter ν is calculated from the moments of the spectrum:

5 $${{\rm{m}}}_{{\rm{n}}}=\,{\int }_{0}^{\infty }\,{{\rm{f}}}^{{\rm{n}}}\,{\rm{S}}({\rm{f}})\,\partial {\rm{f}}$$

where *S*(*f*) is the non-directional energy density spectrum, with $Hs=4\sqrt{{m}_{0}}$.

6 $$\nu =\sqrt{\frac{{m}_{2}{m}_{0}}{{m}_{1}{m}_{1}}}-1$$

where *m*_0_, *m*_1_, and *m*_2_ are the zeroth-, first-, and second-order spectral moments, respectively, calculated from Eq. 5.

For narrow bandwidths ν tends to zero, and the wave energy is concentrated near the spectral peak frequency, as individual waves have similar frequency with differing amplitudes modulated by the wave envelope. Values of ν approaching 1 are due to a broad spectrum, with wave energy distributed over a wide spread of frequencies.

### Correcting for changes in buoy type

Over time, the buoys deployed at each of the 15 locations were upgraded; however, the hull-form and mass of the buoys remains constant, and hence their response to the wave field. The accelerometry also remain constant, and the recorded response is the same across the entire time series. As the buoys were upgraded from the MK1 to the MK2, and then to the MK3 over the course of the time series, there is a change in sample completeness. This results from the MK1 using a shore-based data receiver, whereas the MK2 onwards had on-board data loggers. As with all rare event sampling, rogue wave detection is a function of sampling time; therefore, the rogue wave occurrence count needs to be normalised as a function of sampling.

7 $${\rm{Percentage}}\,{\rm{Rouge}}\,{\rm{Wave}}\,{\rm{Occurance}}=\frac{{\rm{Monthly}}\,{\rm{Rogue}}\,{\rm{Wave}}\,{\rm{Count}}\,\ast \,100}{{\rm{Monthly}}\,{\rm{Wave}}\,{\rm{Count}}}$$

### Estimation of the trend

The aim is to determine the magnitude of a monotonic trend and whether it is statistically significant. A Theil-Sen estimator, an unbiased estimator of the slope in a linear regression, is displayed on the time series of monthly mean resampled data to indicate the trend. The Mann-Kendall (MK) test is a non-parametric test of randomness against trend^30,31^. The presence of a monotonic trend and significance level was assessed using a seasonal MK test, which has been extensively utilised in hydrology research, and was deemed to be the most accurate for assessing trends in the wave data^3^.

The seasonal MK test runs a MK test on each season individually (Boreal Winter: November-January; Spring: February–April; Summer: May–July; Autumn: August–October). The overall statistic is calculated from summing each of the season’s MK statistic. Statistical significance at the 90^th^, 95^th^, and 99^th^ percent level was determined with a two-sided p-value of the overall statistic, less than 0.1, 0.05, and 0.01, respectively. Further sensitivity analysis using bootstrapping is detailed and presented in Supplementary Tables S2 and S3.

### Links with teleconnections

Taking the mean percentage rogue wave occurrence for each season for each year, and plotting against the Multivariate ENSO Index (MEI^21^); yields no relationship^32^.

## Supplementary information

Supplementary Tables
